# Association of Inflammatory and Ischemic Markers with Posterior Segment Parameters in Pseudoexfoliation Syndrome and Glaucoma

**DOI:** 10.3390/jcm14113833

**Published:** 2025-05-29

**Authors:** Muhammed Fatih Satilmaz, Feyzahan Uzun, Hüseyin Findik, Mehtap Atak, Muhammet Kaim, Murat Okutucu, Mehmet Gökhan Aslan

**Affiliations:** 1 Department of Ophthalmology, School of Medicine, Recep Tayyip Erdogan University, 53100 Rize, Turkey; fatihstlmz@gmail.com (M.F.S.); huseyin.findik@erdogan.edu.tr (H.F.); muhammet.kaim@erdogan.edu.tr (M.K.); murat.okutucu@erdogan.edu.tr (M.O.); mehmetgokhan.aslan@erdogan.edu.tr (M.G.A.); 2 Department of Medical Biochemistry, School of Medicine, Recep Tayyip Erdogan University, 53100 Rize, Turkey; mehtap.atak@erdogan.edu.tr

**Keywords:** choroidal thickness, galectin-3, GSH, inflammation, iNOS, MDA, NO, optical coherence tomography angiography, pseudoexfoliative glaucoma, retinal nerve fiber layer, SCUBE-1

## Abstract

**Objective:** This study aimed to investigate the structural, vascular, and biochemical alterations in patients with pseudoexfoliation syndrome (PES) and pseudoexfoliative glaucoma (PXG) and to evaluate the associations between serum biomarkers, the retinal nerve fiber layer (RNFL), choroidal thickness (CT), and vessel density (VD) in these groups. **Methods:** All subjects underwent spectral-domain optical coherence tomography (SD-OCT) and OCT angiography (OCTA) to assess RNFL thickness, CT, and VD. Serum levels of inflammatory and oxidative stress biomarkers—including malondialdehyde (MDA), glutathione (GSH), interleukin-6 (IL-6), nitric oxide (NO), inducible NO synthase (iNOS), galectin-3, and SCUBE-1—were analyzed, and regression and ROC curve analyses were performed to evaluate predictive value and diagnostic performance. **Results:** A total of 80 patients were included and are listed as follows: 25 controls, 30 with PES, and 25 with PXG. There were no significant differences among groups in terms of age or gender. RNFL thickness, CT, and VD were significantly reduced in the PXG group compared to the PES and control groups (*p* < 0.001). PXG patients showed the most pronounced reductions in both peripapillary and macular CT, as well as superficial and deep VD. Serum iNOS, SCUBE-1, galectin-3, and MDA levels were significantly elevated in PXG, while GSH levels were lower (*p* < 0.001); NO levels showed no significant differences. In the PES and PXG groups, several ocular parameters correlated significantly with serum biomarkers, particularly iNOS, MDA, and GSH. Regression analysis in PXG patients identified iNOS and MDA as significant predictors of RNFL thickness and VD. ROC analysis demonstrated that MDA and GSH exhibited the highest diagnostic accuracy among the tested biomarkers for distinguishing PXG patients from controls. **Conclusions:** PXG is associated with significant structural, vascular, and biochemical alterations, including reduced RNFL thickness, choroidal thinning, and decreased VD. Altered serum levels of MDA and GSH were significantly associated with these ocular changes and demonstrated the highest diagnostic accuracy among the biomarkers evaluated. These findings support their potential utility as non-invasive biomarkers for distinguishing PXG from PES and healthy controls and for monitoring disease progression.

## 1. Introduction

Pseudoexfoliation syndrome (PES) is an age-related, multifaceted disorder characterized by the progressive accumulation of abnormal fibrillary extracellular material in ocular and systemic tissues [1]. Although the accumulation predominantly affects the eye, visceral organs including the lungs, liver, kidneys, gall bladder, heart, and cerebral meninges can also be involved [2]. Within the eye, PES primarily leads to the deposition of this material in both the anterior and posterior segments, most commonly at the pupillary edge and on the anterior lens capsule. During pupillary movement, the material is released from the anterior lens capsule through contact between the lens and iris, subsequently accumulating in the trabecular meshwork. This accumulation impairs aqueous humor outflow, elevates intraocular pressure (IOP), and contributes to the development of pseudoexfoliation glaucoma (PXG), a particularly aggressive form of secondary open-angle glaucoma characterized by rapid progression and resistance to conventional treatment [3]. Despite its clinical significance, the underlying mechanisms of PES and its progression to PXG are not yet fully elucidated, but current evidence suggests that ischemic and inflammatory processes may play contributory roles.

Oxidative stress, ischemia, and inflammation are thought to contribute significantly to the pathogenesis of PES. Oxidative stress may be induced by factors such as ultraviolet radiation, aging, and nutritional status [4]. Free radicals generated during oxidative stress can stimulate the secretion of cytokines from various inflammatory cells. These cytokines may contribute to the dysregulated extracellular matrix processes observed in PES and PXG [5]. Malondialdehyde (MDA) is commonly used as a biomarker for oxidative stress, whereas glutathione (GSH) reflects the antioxidative capacity of the system [6]. A reduction in antioxidant levels further exacerbates cytokine release, thereby accelerating the production of pseudoexfoliation material [7]. Studies exploring the relationship between PES and inflammatory markers have demonstrated significant increases in interleukin-6 (IL-6) levels in both the aqueous humor and serum of affected individuals [5,8].

Nitric oxide (NO), a gaseous molecule produced by nitric oxide synthase (NOS) from L-arginine, plays key roles in both normal physiology and disease [9]. Inducible NOS (iNOS), typically inactive, can be triggered in macrophages by bacterial lipopolysaccharides or cytokines. As a vasodilator, NO relaxes smooth muscle and contributes to ocular conditions like cataracts, uveitis, and glaucoma. Elevated NO levels in PES and glaucoma are linked to oxidative stress and trabecular meshwork dysfunction [9].

PES affects not only the eye but also systemic organs and has been identified as an independent risk factor for cardiovascular and cerebrovascular diseases, with its prevalence increasing under ischemic conditions [10]. Galectin-3 and SCUBE-1 are emerging biomarkers associated with ischemic and atherosclerotic conditions, including acute ischemic stroke, embolism, and coronary syndrome [11,12]. Recent studies have linked elevated galectin-3 levels to various ocular conditions, such as retinal degeneration, diabetic retinopathy, dry eye, and glaucoma [13,14,15]. Additionally, increased SCUBE-1 levels have been observed in the aqueous humor of patients with PES [16].

The choroid, one of the most vascularized tissues in the body, is primarily supplied by the long and short ciliary arteries, with additional contributions from the anterior ciliary arteries. Choroidal thickness, an indicator of blood flow, is reduced in PES, which may lead to the narrowing or obstruction of the choroidal vessels [17,18]. Evaluating the peripapillary choroid is crucial for understanding the pathogenesis of PES and PXG.

Optical coherence tomography angiography (OCT-A) is a novel, noninvasive imaging technique that enables visualization of retinal microcirculation and provides quantitative analysis of the peripapillary and macular microvasculature. Given the hypothesis that compromised vascular nutrition contributes to the pathogenesis of PXG, OCT-A has become increasingly utilized in patients with PES and PXG [19].

This study aims to investigate the relationship between PES/PSX and alterations in choroidal thickness, peripapillary vascular structures, and oxidative stress markers. By leveraging advanced imaging modalities, including OCT-A, alongside the analysis of oxidative stress and inflammatory biomarkers, we seek to elucidate the underlying mechanisms that contribute to the pathogenesis of PES and its progression to PXG.

## 2. Materials and Methods

This prospective observational study was conducted at a tertiary university hospital, and ethical approval was obtained from the Recep Tayyip Erdogan University Ethics Committee. All study procedures adhered to the principles outlined in the Declaration of Helsinki, and all participants provided written informed consent.

### 2.1. Patient Selection

The diagnosis of PXG was established based on the presence of an open angle on gonioscopy, IOP >21 mmHg in patients not previously using antiglaucomatous medications, or IOP <21 mmHg under medical treatment. Additional diagnostic criteria included typical glaucomatous appearance of the optic disc (e.g., neuroretinal rim thinning or notching) on fundus examination, glaucomatous retinal nerve fiber layer (RNFL) thinning on optical coherence tomography (OCT), characteristic visual field defects confirmed by standard automated perimetry, and the presence of pseudoexfoliation material observed during gonioscopy. To ensure uniformity, only patients with early to moderate-stage PXG were included, based on the Hodapp–Parrish–Anderson classification [20], defined as mean deviation values better than −12 dB. Although the duration of PXG was estimated based on medical records, with inclusion limited to patients diagnosed within the past five years, it is important to acknowledge that glaucoma is a clinically silent and slowly progressive disease. As such, the onset of glaucoma may precede clinical detection by several years and, in some cases, be identified incidentally during routine ophthalmological evaluations, making it difficult to determine the exact timing of disease onset with certainty.

This study included 30 eyes from 30 patients with PES, as well as 25 eyes from 25 patients in the PXG and control groups. For cases of unilateral involvement, the affected eye was selected, whereas in cases of bilateral involvement, the right eye was chosen for analysis. Participants were categorized into the following three groups: those diagnosed with PES in at least one eye following a comprehensive ophthalmological examination, those diagnosed with PXG in at least one eye, and a control group comprising ocularly healthy individuals, except for the presence of cataracts. During the examinations, RNFL thickness, OCT, and OCT-A images were obtained. Parameters such as RNFL thickness, choroidal thickness (CT), and vessel density (VD) were measured from these images. Additionally, oxidative, ischemic, and inflammatory markers were analyzed from blood samples.

### 2.2. Exclusion Criteria

Patients in the PES and PXG groups who had undergone retinal photocoagulation, glaucoma laser procedures (e.g., laser trabeculoplasty), had chorioretinal diseases, or had a history of any prior ocular surgery were excluded from this study to minimize potential confounding effects on the study outcomes. To ensure the reliability of systemic inflammatory, ischemic, and oxidative stress marker analyses, patients in the PES and PXG groups were required to have no additional systemic diseases. Similarly, control group participants, who were ocularly healthy except for the presence of cataracts, were also free of systemic diseases. To avoid interference with OCT-A imaging, individuals with spherical refractive errors exceeding ±5 diopters or cylindrical refractive errors exceeding ±3 diopters were excluded from the study.

### 2.3. OCT Imaging

**Figure 1:**
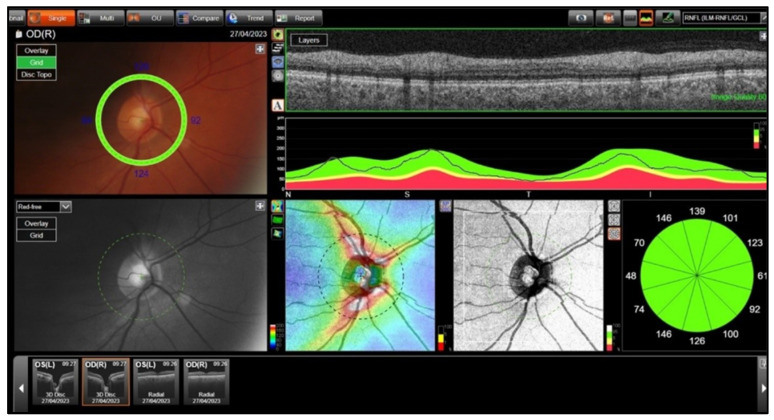
The technique of RNFL measurements.

Choroidal and RNFL thicknesses were measured using swept-source OCT and OCT-A (DRI OCT Triton; Topcon, Tokyo, Japan). All participants underwent pupillary dilation prior to imaging, and all OCT and OCTA measurements were performed using the same imaging device by a single, experienced technician who was masked to the clinical data of the participants. Standardized imaging protocols were followed to ensure consistency across all scans. Images with motion artifacts, segmentation errors, or a signal strength index below the device’s recommended threshold were excluded from the analysis. To minimize the impact of diurnal variations, imaging sessions were scheduled within a fixed timeframe each day (10:00–12:00 a.m.). RNFL thickness and peripapillary CT were assessed using a standard 360-degree, 3.4 mm diameter circle scan centered on the optic disc. RNFL thickness was recorded in four automatically measured quadrants (Figure 1).

**Figure 2:**
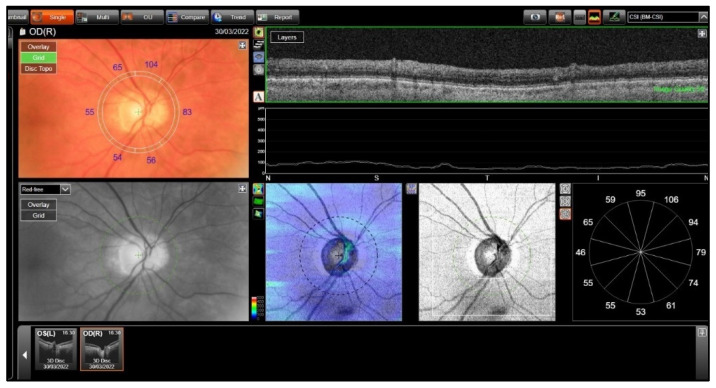
The technique of peripapillary choroidal thickness measurements.

CT measurements were performed in the following six quadrants of the peripapillary region: temporal, inferotemporal, inferonasal, nasal, superonasal, and superotemporal. These measurements were obtained in the fast RNFL mode by selecting the GRID mode on the OCT device. The device provided automatically measured choroidal average thickness by selecting the Bruch’s membrane–internal scleral interface (BM-CSI) layer from the results (Figure 2).

**Figure 3:**
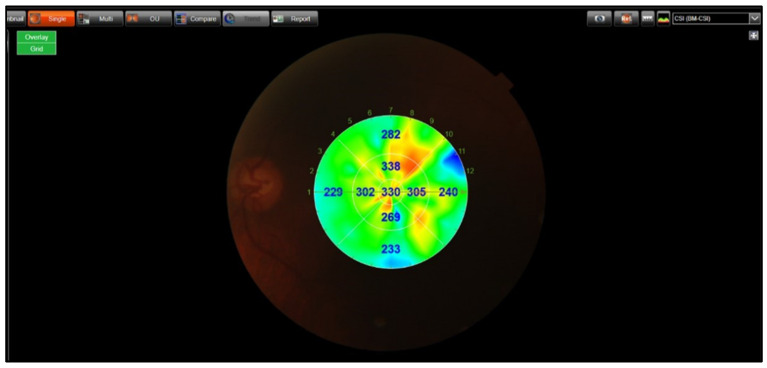
The technique of macular choroidal thickness measurements.

For macular CT, a choroidal map derived from 12 mm radial scans was utilized. This map automatically displayed the subfoveal choroidal thickness in nine subfields, along with the average thickness of these subfields based on the Early Treatment Diabetic Retinopathy Study (ETDRS) guideline. The average choroidal thicknesses were recorded in relation to the fovea, including subfoveal (1 mm diameter), inner ring (3 mm diameter), and outer ring (6 mm diameter) areas (Figure 3).

### 2.4. OCT-A Imaging

**Figure 4:**
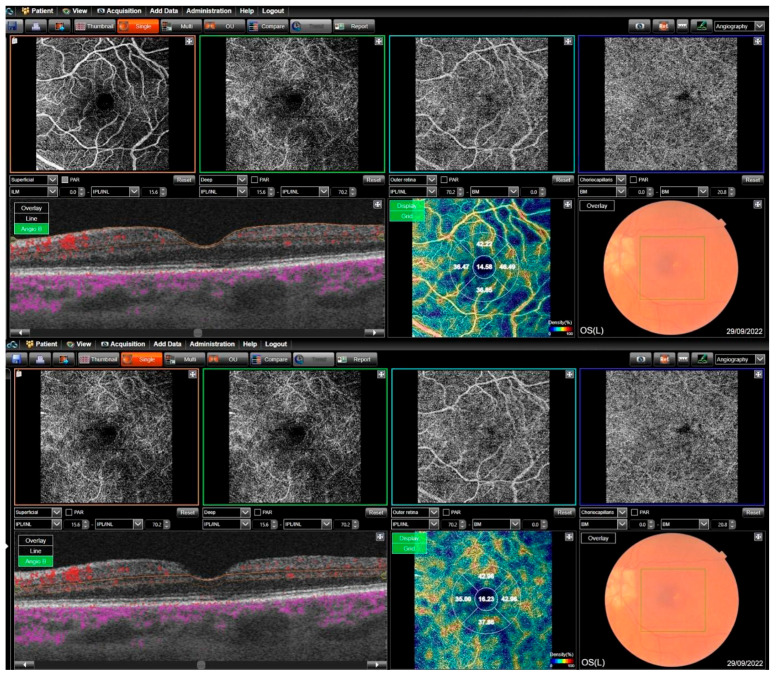
The technique of superficial (**top**) and deep (**bottom**) vessel density measurements.

Two retinal layers, the superficial retinal layer and the deep retinal layer, were analyzed for VD. A 6 × 6 mm^2^ field map was used for all images. In accordance with the device specifications, the inner boundary of the superficial retinal layer was defined as 2.6 µm below the internal limiting membrane (ILM), and the outer boundary was set at 15.6 µm below the inner plexiform layer (IPL). The inner border of the deep retinal layer was set at 15.6 µm, with the outer border at 70.2 µm below the IPL. Thus, the area between 2.6 and 15.6 µm from the retinal surface was considered the superficial retinal layer, while the area between 15.6 and 70.2 µm from the surface was classified as the deep retinal layer. Vessel densities were measured in the foveal region (1 mm diameter central area) and the parafoveal region (1–3 mm diameter ring). The parafoveal region was further divided into four sections, each covering 90 degrees (nasal, inferior, superior, and temporal sectors) (Figure 4).

### 2.5. Measurements of Inflammatory and Ischemic Markers

Venous blood samples were collected after an overnight fast of at least 12 h, between 9:00 a.m. and 12:00 p.m., to minimize metabolic and diurnal variations. Samples were drawn into appropriate collection tubes based on biomarker requirements, avoiding anticoagulants such as dipotassium EDTA and sodium fluoride/disodium EDTA, which could interfere with assay results. Plasma was separated within one hour of collection and stored at −80 °C until analysis.

### 2.6. Malondialdehyde (MDA)

The levels of MDA, a product of lipid peroxidation, were measured spectrophotometrically based on the absorbance of the pink complex formed by the reaction of MDA with thiobarbituric acid (TBA) solution (Thiobarbituric Acid, Cayman Chemical Company, Ann Arbor, MI, USA) [21]. The reaction mixture was heated, and the absorbance of the resulting complex was measured at a wavelength of 532 nm. A standard curve was generated using MDA solutions at concentrations of 40, 20, 10, 5, and 2.5 nmol/mL. Serum MDA concentrations were calculated using the standard curve, and the results were expressed as nmol/mL of MDA.

### 2.7. Glutathione (GSH)

The serum levels of GSH were determined using a spectrophotometric method based on the yellow color produced by free sulfhydryl groups in reaction with Ellman’s reagent (5,5′-Dithiobis-(2-nitrobenzoic acid), Sigma-Aldrich, St. Louis, MO, USA) [22]. A standard curve was generated by the serial dilution of a 1 mM stock solution of L-glutathione (reduced), with final concentrations of 0.5, 0.25, 0.125, and 0.0625 mM. Absorbance was measured at a wavelength of 412 nm using a spectrophotometer. GSH concentrations in the serum samples were calculated using the standard curve, and the results were expressed in millimolar (mM) units.

### 2.8. Nitric Oxide (NO)

Serum NO levels were measured using a commercially available colorimetric assay kit (ELASCCIENCE, Cat. No: E-BC-K035-M, Lot: NM0942621369). Serum, standards, or bi-distilled water (300 µL) were mixed with 200 µL of Reagent 1 and 100 µL of Reagent 2, vortexed, and incubated at room temperature for 15 min. After centrifugation at 3100 g for 10 min, 160 µL of the supernatant was combined with 80 µL of Chromogenic Reagent, shaken for 2 min, and incubated at room temperature for 15 min. Absorbance was measured at 550 nm. A sodium nitrite standard curve (1–0.0312 mmol/L) was used for calculations.

### 2.9. SCUBE-1, Galectin-3,iNOS, and IL-6

Serum levels of SCUBE-1, galectin-3, iNOS, and IL-6 were measured using commercially available sandwich ELISA kits (SCUBE-1: Cat. No. E-EL-H5405; galectin-3: E-EL-H1470; i-NOS: E-EL-H0753; IL-6: E-EL-H6156). Standards (100 µL) and serum samples (100 µL) were pipetted into wells pre-coated with the respective antibodies and incubated at 37 °C for 90 min. After removal of the liquid, 100 µL of biotin-labeled antibody was added and incubated for 60 min at 37 °C. Following three washes, 100 µL of HRP conjugate was added and incubated for 30 min at 37 °C. Wells were then washed five times, and 90 µL of substrate reagent was added, incubated at 37 °C for 15 min, and protected from light. The reaction was stopped with 50 µL of stop solution. Absorbance was measured at 450 nm using an ELISA reader. Concentrations were calculated in ng/mL using standard curves generated from the absorbance values of known standards.

### 2.10. Statistical Analysis

A priori sample size estimation was performed using G*Power software (version 3.1.9.7, Düsseldorf, Germany) to ensure adequate statistical power for detecting differences among the three study groups. Based on a one-way ANOVA with an assumed effect size of 0.40, a significance level of 0.05, and a desired power of 0.85, the required total sample size was calculated to be 72 participants. The final study sample of 80 eyes met this requirement, confirming that the study was sufficiently powered.

All statistical analyses were conducted using IBM SPSS Statistics for Windows, version 29.0 (IBM Corp., Armonk, NY, USA). The Shapiro–Wilk test was used to assess the normality of continuous variables. As most variables were not normally distributed, non-parametric tests were employed. Categorical variables were presented as frequencies and percentages, while continuous variables were summarized using means, standard deviations, medians, and minimum–maximum values.

Group comparisons for continuous variables were performed using the Kruskal–Wallis H test, while categorical variables were analyzed using the Chi-Square test or Fisher’s Exact test where appropriate. When significant differences were found, post hoc comparisons were conducted using the Mann–Whitney U test, and Bonferroni correction was applied to adjust for multiple pairwise comparisons. Friedman’s Two-Way Analysis of Variance by Ranks was used for evaluating repeated dependent measurements. Spearman’s correlation analysis was employed to examine relationships between serum biomarkers and posterior segment parameters. Given the multiple correlations tested, a Bonferroni-adjusted significance threshold was applied to reduce the risk of type I error, and only correlations that remained significant after adjustment were interpreted.

Linear regression analysis was used to evaluate the predictive value of serum biomarker levels on RNFL thickness, peripapillary and macular choroidal thickness, and superficial and deep vessel densities. Receiver operating characteristic (ROC) curve analysis was conducted to assess the diagnostic accuracy of serum biomarkers, and the area under the curve (AUC) was calculated. A two-sided *p* value of <0.05 was considered statistically significant unless otherwise specified.

## 3. Results

A total of 80 patients were included in the study. Of these, 31.3% (*n* = 25) comprised the control group, 37.5% (*n* = 30) were diagnosed with PES, and 31.3% (*n* = 25) were diagnosed with PXG. The patients’ ages ranged from 55 to 85 years, with a mean age of 73 years. Male patients accounted for 52.5% (*n* = 42) of the cohort, while female patients comprised 47.5% (*n* = 38). There were no statistically significant differences among the groups with respect to age or gender distribution, intraocular pressure, and central corneal thickness (Table 1).

### SD: Standard Deviation

The analysis of the distribution of RNFL thickness and vessel density (VD) among the groups revealed significant differences in both RNFL thickness and VD values (*p* < 0.001). The mean RNFL thickness was 102.1 ± 5.2 µm in the control group, was 100.9 ± 9.8 µm in the PES group, and was significantly lower at 62.7 ± 11.2 µm in the PXG group. Similarly, RNFL thickness in the inferior, superior, nasal, and temporal regions was markedly reduced in the PXG group compared to the other groups (*p* < 0.001). The distribution of peripapillary and macular choroidal thickness measurements revealed significant differences. Peripapillary choroidal thickness was significantly reduced in the PXG group compared to the PES and control groups across all measured regions (*p* < 0.001). For example, the mean overall peripapillary choroidal thickness was 151.2 ± 12.9 µm in the control group, 128.6 ± 31.1 µm in the PES group, and 94.1 ± 26.5 µm in the PXG group. Similarly, macular choroidal thickness showed a significant reduction in the PXG group compared to the control and PES groups (*p* < 0.001), with subfoveal choroidal thickness measuring 285.84 ± 17.16 µm in controls, 253.13 ± 46.25 µm in PES, and 223.76 ± 31.30 µm in PXG.

Regarding vessel density, both superficial and deep vessel density measurements were significantly lower in the PXG group compared to the other groups (*p* < 0.001). Superficial vessel density was measured as 43.5 ± 2.9% in the control group, 38 ± 2.8% in the PES group, and 33.3 ± 1.5% in the PXG group. Likewise, fovea, inferior, superior, nasal, and temporal values were significantly reduced in the PXG group (*p* < 0.001). However, no statistically significant differences were observed in deep vessel density in the superior, nasal, and temporal regions among the groups (*p* > 0.05).

These findings indicate a pronounced reduction in both RNFL thickness and vessel density in the PXG group, while the PES group exhibited less pronounced changes compared to the control group (Table 2).

The analysis of serum biomarkers also demonstrated significant differences among the groups. iNOS levels were highest in the PXG group (8 ± 1.1) compared to the PES (6 ± 0.8) and control (4 ± 1.5) groups (*p* < 0.001). SCUBE-1 and galectin-3 levels were significantly elevated in the PXG group, while MDA levels followed a similar pattern. Conversely, GSH levels were significantly lower in both the PES and PXG groups compared to controls (*p* < 0.001). Notably, no significant differences were observed for NO levels across the groups (*p* = 0.821).

These findings highlight significant alterations in serum biomarker levels, indicating pronounced vascular and biochemical changes associated with the disease (Table 3).

In the PES group, the correlations between age, serum biomarkers, and ocular measurements demonstrated varied relationships. For RNFL thickness, iNOS exhibited a moderately positive correlation with average RNFL thickness (r = 0.336, *p* = 0.069), though not statistically significant. A statistically significant negative correlation was observed between nasal RNFL thickness and iNOS (r = −0.453, *p* = 0.012), as well as with SCUBE-1 (r = −0.383, *p* = 0.037).

Peripapillary choroidal thickness showed a positive but non-significant correlation with biomarkers, except for a significant association between inferonasal choroidal thickness and NO (r = 0.439, *p* = 0.015). Regarding macular choroidal thickness, subfoveal measurements correlated positively with GSH (r = 0.441, *p* = 0.015) and negatively with galectin-3 (r = −0.450, *p* = 0.013) and MDA (r = −0.457, *p* = 0.011) in outer temporal regions.

Superficial vessel density in the foveal region negatively correlated with MDA (r = −0.538, *p* = 0.002), while deep vessel density in the superior quadrant negatively correlated with MDA (r = −0.451, *p* = 0.012) and galectin-3 (r = −0.398, *p* = 0.029).

These findings suggest a potential interplay between inflammatory and oxidative stress markers, choroidal parameters, and vessel density in PES patients. However, the majority of correlations were non-significant, indicating the need for further studies to elucidate these relationships (Table 4).

In the PXG group, the correlations between age, serum biomarkers, and ocular measurements were analyzed (Table 5). A statistically significant negative correlation was observed between nasal peripapillary choroidal thickness and IL-6 levels (r = −0.472, *p* = 0.017). Similarly, superonasal peripapillary choroidal thickness showed a strong positive correlation with GSH levels (r = 0.651, *p* = 0.002) and a significant negative correlation with MDA levels (r = −0.644, *p* = 0.001). Inferonasal peripapillary choroidal thickness also demonstrated a strong positive correlation with NO levels (r = 0.624, *p* = 0.001). Regarding macular choroidal thickness, inner temporal and outer temporal regions exhibited significant negative correlations with age (r = −0.459, *p* = 0.021; r = −0.448, *p* = 0.025, respectively). Additionally, subfoveal VD at the deep vascular plexus correlated negatively with SCUBE-1 levels (r = −0.454, *p* = 0.023), and temporal VD in the deep vascular plexus was inversely associated with MDA (r = −0.507, *p* = 0.010). These findings suggest that specific serum biomarkers and age may influence structural and vascular ocular parameters in PXG (Table 5).

The influence of serum biomarker levels on various ocular measurements was analyzed using regression models in the PXG group (Table 6). For RNFL thickness, iNOS levels showed a significant negative association (β = −5.816, *p* = 0.031), with the model explaining 47.1% of the variance (R^2^ = 0.471, *p* = 0.049). Regarding superficial vessel density, iNOS (β = −0.786, *p* = 0.003) and MDA (β = −0.246, *p* = 0.005) levels were significant negative predictors, and the model accounted for 66.2% of the variance (R^2^ = 0.662, *p* = 0.002). For deep vessel density, significant negative associations were observed with iNOS (β = −0.977, *p* = 0.012) and MDA (β = −0.261, *p* = 0.037), with the model explaining 72.6% of the variance (R^2^ = 0.726, *p* = 0.022). These findings suggest that iNOS and MDA may be associated with vascular and structural ocular changes observed in PXG patients.

**Figure 5:**
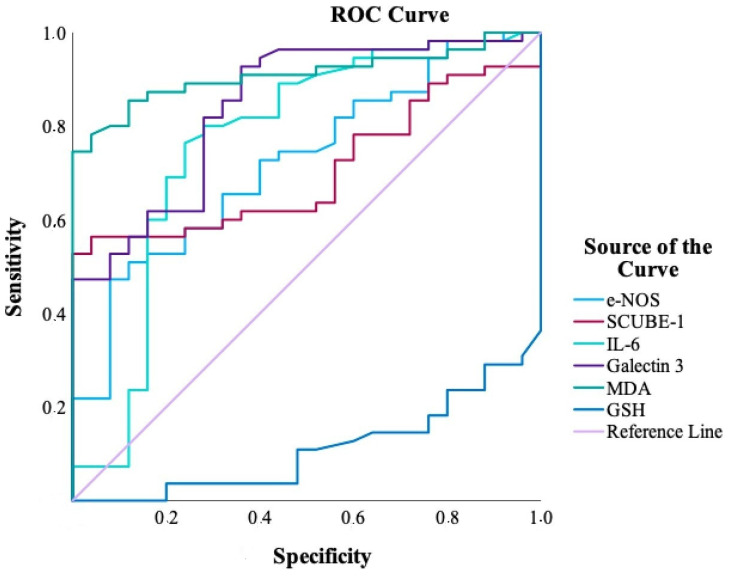
Diagnostic performance of inflammatory and oxidative stress markers in pseudoexfoliation glaucoma: ROC curve analysis.

Receiver operating characteristic (ROC) analysis was conducted to assess the diagnostic performance of serum biomarkers in distinguishing PXG patients from controls (Figure 5, Table 7). Among the biomarkers evaluated, MDA demonstrated the highest diagnostic accuracy, with an AUC of 0.909 (95% CI: 0.846–0.973), a sensitivity of 74.5%, and a perfect specificity of 100.0%, indicating excellent discriminative ability. GSH followed closely with an AUC of 0.890 (95% CI: 0.822–0.958), sensitivity of 70.9%, and specificity of 96.0%. Galectin-3 showed high sensitivity (92.7%) but lower specificity (64.0%), with an AUC of 0.843, reflecting moderate diagnostic value. IL-6 yielded an AUC of 0.766, indicating fair performance, with balanced sensitivity (76.4%) and specificity (76.0%). In contrast, iNOS and SCUBE-1 displayed lower AUCs (0.723 and 0.705, respectively), with limited sensitivity (47.3% and 52.7%), despite high specificity (92.0% and 100.0%). These results, visually supported by the ROC curves (Figure 5), underscore MDA and GSH as the most promising serum biomarkers for differentiating PXG from controls.

## 4. Discussion

This study demonstrated significant differences in retinal nerve fiber layer (RNFL) thickness, choroidal parameters, vessel density (VD), and serum biomarker levels among patients with pseudoexfoliation syndrome (PES), pseudoexfoliation glaucoma (PXG), and healthy controls. Notably, RNFL thickness and VD were markedly reduced in PXG patients, particularly in the inferior, superior, nasal, and temporal regions. Similarly, both peripapillary and macular choroidal thickness were significantly lower in the PXG group, reflecting structural alterations associated with disease progression. Serum biomarker analysis revealed elevated levels of iNOS, SCUBE-1, galectin-3, and MDA in PXG patients, while GSH levels were significantly reduced. Regression analysis suggested that iNOS and MDA levels were significantly associated with alterations in vascular and structural ocular parameters observed in PXG patients. Additionally, ROC curve analysis demonstrated that MDA and GSH exhibited the highest diagnostic accuracy for differentiating PXG from controls, based on their superior AUC, sensitivity, and specificity values. These findings suggest a potential link between oxidative stress markers and structural, as well as vascular, alterations in PXG, supporting their relevance in the underlying pathophysiological mechanisms of the disease.

Demircan et al. observed that the RNFL was significantly thinner in the PXG group compared to the PES and control groups. Similarly, choroidal thickness was significantly reduced in the PXG and PES groups compared to the control group. They concluded that PES may compromise choroidal circulation by accumulating in choroidal vessels, and the thinner choroid in the PXG group suggests that ischemia influences the progression of PES and contributes to glaucoma development [23]. While a number of studies have consistently demonstrated a significant reduction in RNFL and choroidal thickness in PXG patients compared to controls [24,25], findings regarding the PES group remain variable. Turan Vural et al. reported that PES is linked to overall subfoveal choroidal thinning and a significant reduction in ocular perfusion pressure [26]. Toptan et al. reported that RNFL thickness was significantly lower in both PXG and PES eyes compared to controls, while macular choroidal thickness was significantly reduced only in the PXG group, with no significant difference between PES and controls. They observed that a longer duration of exposure to pseudoexfoliation material is associated with greater reductions in RNFL and choroidal thickness. [27]. Yüksel et al. [28] identified significant RNFL thinning in the inferior and superior quadrants in PES patients, whereas Ozmen et al. [29] reported significant thinning in the inferior quadrant and average RNFL thickness. In our study, RNFL thickness in the PES group was not significantly different from that in the control group. However, both average peripapillary and subfoveal macular choroidal thicknesses were lower in the PES group compared to controls. The discrepancies in RNFL thickness between the PES and control groups across different studies may be attributed to variations in disease stage and duration among the patient cohorts.

It has been demonstrated that the accumulation of pseudoexfoliation material in the vessel walls disrupts retrobulbar blood flow in both PES and PXG groups [30,31]. In our study, it was observed that the peripapillary choroidal thickness in the nasal, superonasal, and inferonasal regions was thinner in PXG patients compared to the other groups. Additionally, significant differences were found in the temporal, superotemporal, and inferotemporal regions among all groups (Table 1). The significant thickness differences in quadrants between the PES and control groups are consistent with the early course of glaucoma, suggesting that the vascular impairment of the optic disc head is already present in the PES group [32]. A recent study by Un et al. also reported that the peripapillary choroidal thickness was thinner in the PES group compared to healthy controls. They found no significant difference between the non-affected eyes of asymmetrically involved patients and the healthy control group, indicating that PES material alone affects the peripapillary choroidal thickness [17].

In the macular choroidal thickness, significant differences were observed between all groups in the subfoveal, inner nasal, and outer nasal regions (Table 1). These results suggest impaired choroidal perfusion in the macular region, particularly in the nasal area close to the optic disc. Unlike other retinal tissues, the fovea is not supplied by the central retinal artery but by the choroid [6]. Therefore, in conditions where choroidal perfusion is compromised, the choroidal thickness in the fovea may become thinner. Dursun et al. [33] reported thinner subfoveal choroidal thickness in the PES and PXG groups, while Bayhan et al. found no significant differences in subfoveal and temporal choroidal thickness measurements between PXG patients and controls; however, they observed thinning in the nasal choroid in PXG cases [34]. Çınar et al. reported that subfoveal choroidal thickness was significantly lower and foveal avascular zone area was significantly enlarged in the PES group compared to control eyes [35].

Studies on vascular density have also been conducted in PES and PXG groups. In our study, the superficial vascular density progressively decreased across all quadrants in the PES and control groups, with the lowest values observed in the PXG group. In terms of deep vascular density, the PXG group exhibited lower values in the foveal and inferior quadrants compared to the other two groups (Table 2). *Çınar* et al. found that superficial vascular density was reduced in all quadrants of the PES group compared to the control group [35], while Rebolleda et al. reported lower capillary density in the PXG group compared to primary open-angle glaucoma and controls [36]. In another study, a correlation between reduced vascular density and thinner RNFL thickness was observed in the PXG group, thus highlighting the relationship between structural and vascular parameters [37].

The pathogenesis of PES involves a complex interplay of genetic, environmental, and biochemical factors, leading to the accumulation of abnormal fibrillar material in ocular tissues [4]. Recent studies have emphasized the role of inflammatory and ischemic biomarkers in both the development of PES and its progression to PXG [38,39]. In studies conducted with oxidative stress biomarkers, Cumurcu et al. found that, in the PES group, serum total oxidative capacity (TOC) was high, while total antioxidant capacity (TAC) was low [40]. Yağcı et al. reported elevated MDA levels and reduced superoxide dismutase activity in the PES group, suggesting the role of oxidative stress in the pathogenesis of PES [41]. Yaz et al. concluded that elevated MDA levels indicate the significance of lipid peroxidation in the development of PES, while higher GSH levels may reflect a compensatory response to oxidative stress in PXG [7]. Our results showed that MDA was elevated and GSH was reduced in the patient groups. This indicates an imbalance in the oxidant–antioxidant balance in PES and PXG. Correlation analyses revealed a linear relationship between the measurements and GSH, while an inverse relationship was observed with MDA. In regression analyses, it was found that MDA had a significant effect in reducing RNFL and vessel density values in the PXG group.

IL-6 is a pro-inflammatory cytokine involved in inflammation and angiogenesis in the conjunctiva, cornea, iris, retina, and orbit [42]. In a study by Zenkel et al., IL-6 and IL-8 levels were found to be three times higher in the aqueous humor of early-stage PES patients compared to the control group [5]. However, in the late stages of PES/PXG, these cytokine levels were not significantly different from those in the control group. Yıldırım et al. reported elevated serum IL-6 levels in PES patients [8]. Vulovic et al. indicated that, while IL-6 plays a role in the early stages of PES and PXG, TNF-α and IL-17 are the primary drivers of inflammation activation in both the early and late stages of the disease [43]. In our study, we observed higher serum IL-6 levels in the PES group compared to the other groups, consistent with previous findings.

NO plays a crucial role in vascular homeostasis, neuroprotection, and oxidative stress regulation [44], and its dysregulation has been implicated in the pathogenesis of PES and PXG. While some studies have reported similar serum NO levels among PES, PXG, and control groups [45], differences have been observed in aqueous humor NO concentrations. Borazan et al. [46] found elevated NO levels in the aqueous humor not in the plasma of PES and PXG patients compared to controls, whereas Kotikoski et al. reported reduced nitrite + nitrate levels in the aqueous humor of pseudoexfoliation patients [47]. These inconsistencies may be due to the inherent challenges of NO measurement, including its short half-life, rapid interaction with free radicals, and strict storage requirements. To overcome these limitations, we analyzed both NO and its inducible form, iNOS, which is the only actively stimulated form of NO. In the eye, iNOS is upregulated in response to oxidative and inflammatory stimuli and has been shown to disrupt vascular homeostasis, increase blood–retina barrier permeability, and induce the apoptosis of retinal ganglion cells through excess nitric oxide production [48]. Elevated iNOS expression has been reported in ocular tissues such as the trabecular meshwork, optic nerve head, and retina in glaucomatous eyes, contributing to neurodegeneration and impaired aqueous outflow [49]. Our study revealed significantly higher serum iNOS levels in the PXG group compared to other groups, despite no significant differences in NO levels. This suggests that iNOS may play a role in the inflammatory and oxidative processes contributing to PXG progression, potentially explaining why previous studies focusing solely on NO did not find statistically significant differences.

Galectin-3 and SCUBE-1 have been implicated in the pathogenesis of PES and PXG through their roles in inflammation, fibrosis, and vascular remodeling. Galectin-3, a β-galactoside-binding lectin, is involved in oxidative stress, immune modulation, and extracellular matrix turnover, which are processes that contribute to the abnormal fibrillar material accumulation characteristic of PES [50]. Elevated galectin-3 levels have been associated with increased fibrosis and inflammation in ocular tissues, potentially accelerating the progression to PXG [14]. SCUBE-1, a cell surface protein linked to platelet activation and endothelial function, is considered a marker of thrombotic and ischemic events. Altered SCUBE-1 levels in PES and PXG suggest its involvement in microvascular dysfunction and impaired ocular blood flow, further contributing to glaucomatous damage [16]. In our study, serum SCUBE-1 and galectin-3 levels were found to be elevated in the PXG group compared to the other two groups. These biomarkers offer insight into the ischemia-related mechanisms underlying PES and PXG and may serve as potential targets for early diagnosis and therapeutic intervention.

In our study, the diagnostic performance analysis using ROC curves revealed that MDA and GSH had the highest sensitivity and specificity in distinguishing PXG patients from controls. Previous studies have highlighted that both MDA and GSH play systemic roles in oxidative stress and inflammation, particularly in cardiovascular and neurodegenerative diseases [51,52]. However, their relevance in ocular disease is increasingly being recognized [53]. Oxidative stress is implicated in the pathogenesis of PXG, with elevated MDA levels—an indicator of lipid peroxidation and membrane damage—reported in both serum and aqueous humor [54]. Concurrently, a reduction in GSH, a key antioxidant, reflects impaired antioxidant defense mechanisms in PXG [6]. Although MDA and GSH are not exclusively specific to ocular tissues, the exclusion of participants with known systemic diseases in our study helps minimize potential confounding and supports the interpretation that the observed alterations in their levels among PXG patients may be associated with ocular-specific pathophysiological processes.

This study has several limitations. First, the relatively small sample size may affect the generalizability of our findings. The exclusion of patients with systemic diseases—such as diabetes mellitus, hypertension, and cardiovascular disease—was intentionally implemented to minimize systemic confounding and isolate the ocular-specific effects of pseudoexfoliation and glaucomatous changes. However, this strict selection criterion particularly limited the recruitment of participants in the PXG group and may have biased the cohort toward healthier individuals, thereby limiting the applicability of the results to the general PXG population, in which vascular comorbidities are common. The potential systemic or local effects of topical antiglaucoma medications on serum biomarker levels or ocular structural and vascular parameters were not specifically accounted for, which may represent a confounding factor in the interpretation of the results. Additionally, while systemic biomarker levels were analyzed, assessing these markers in the aqueous humor could have provided a more direct evaluation of local ocular changes associated with PES and PXG. Future studies involving larger and more heterogeneous populations, including those with systemic vascular conditions, are warranted to validate and expand upon our findings.

## 5. Conclusions

PES is a complex, multifactorial disorder that involves both ocular and systemic alterations, contributing to disease progression and increased risk of glaucoma. This study underscores the potential involvement of inflammatory, ischemic, and oxidative stress pathways in the pathophysiology of PES and its progression to pseudoexfoliation glaucoma (PXG). Altered serum levels of biomarkers, such as MDA, GSH, iNOS, SCUBE-1, and galectin-3, support the contribution of oxidative stress and ischemic mechanisms to PXG development. While these findings offer meaningful insights, further large-scale, longitudinal studies—incorporating aqueous humor analysis and advanced ocular imaging—are warranted to validate the prognostic significance of these biomarkers and evaluate their therapeutic potential. A deeper understanding of these molecular mechanisms may facilitate earlier diagnosis and enable the development of more targeted treatment strategies in the management of PES and PXG. 

## Figures and Tables

**Table 1:** Demographic and clinical characteristics of the study participants across control, PES, and PXG groups.

	Control (1) (*n* = 25)	PES (2) (*n* = 30)	PXG (3) (*n* = 25)	*p*-Value
Age (years) (mean ± SD)	72 ± 7.2	74 ± 6.9	73 ± 7.1	0.46
Gender (*n*) (male/female)	12/13	16/14	14/11	0.84
Intraocular pressure (mmHg) (mean ± SD)	15.8 ± 3.5	16.2 ± 3.4	17.02 ± 3.1	0.41
Central corneal thickness (mean ± SD)	567.1 ± 32.3	563.05 ± 22.7	561.3 ± 25.1	>0.05 *

* *p* < 0.05; statistically significant.

**Table 2:** Comparison of the retinal nerve fiber layer, choroidal thickness, and retinal vessel density parameters among control, pseudoexfoliation syndrome (PES), and pseudoexfoliation glaucoma (PXG) groups.

	Control (1)	PES (2)	PXG (3)	*p*-Value	
	(*n* = 25)	(*n* = 30)	(*n* = 25)		
RNFL					
Average					
Mean ± SD	102.1 ± 5.2	100.9 ± 9.8	62.7 ± 11.2	<0.001 *	1–3; 2–3
Median (min–max)	103 (92–110)	100.5 (84–129)	62 (41–87)		
Inferior					
Mean ± SD	125.2 ± 13.4	125.8 ± 17.2	70.6 ± 20.7	<0.001 *	1–3; 2–3
Median (min–max)	125 (101–151)	126 (93–153)	66 (39–114)		
Superior					
Mean ± SD	120.8 ± 14.3	118.6 ± 15.3	72.9 ± 14.8	<0.001 *	1–3; 2–3
Median (min–max)	121 (93–148)	115.5 (93–149)	72 (54–120)		
Nasal					
Mean ± SD	84.7 ± 12	85.5 ± 12.7	57.1 ± 13.1	<0.001 *	1–3; 2–3
Median (min–max)	85 (53–102)	84 (59–110)	57 (33–85)		
Temporal					
Mean ± SD	74.6 ± 13.9	72.7 ± 14.5	51.3 ± 12.3	<0.001 *	1–3; 2–3
Median (min–max)	72 (52–115)	70 (53–113)	50 (30–83)		
Peripapillary Choroidal Thickness					
Average					
Mean ± SD	151.2 ± 12.9	128.6 ± 31.1	94.1 ± 26.5	<0.001 *	1– 2; 1–3; 2–3
Median (min–max)	147.8 (134–185)	124.2 (85–196)	87.5 (58–171)		
Nasal					
Mean ± SD	137.88 ± 15.42	133.3 ± 30.98	108.24 ± 32.24	<0.001 *	1–3; 2–3
Median (min–max)	139 (102–172)	125 (80–200)	100 (51–162)		
Superonasal					
Mean ± SD	146.56 ± 18.82	135.3 ± 23.34	106.84 ± 27.30	<0.001 *	1–3; 2–3
Median (min–max)	142 (110–176)	133.5(101–186)	96 (75–188)		
Superotemporal					
Mean ± SD	160.08 ± 12.84	129.93 ± 30.79	99.56 ± 35.71	<0.001 *	1–2; 1–3; 2–3
Median (min–max)	160 (131–182)	126.5 (78–222)	98 (51–215)		
Temporal					
Mean ± SD	156.32 ± 9.43	128.7 ± 31.59	102.72 ± 23.57	<0.001 *	1–2; 1–3; 2–3
Median (min–max)	155 (140–179)	128 (74–206)	98 (70–164)		
Inferotemporal					
Mean ± SD	141.88 ± 17.26	116.6 ± 35.03	88.08 ± 21.91	<0.001 *	1–2; 1–3; 2–3
Median (min–max)	136 (111–182)	118 (51–205)	85 (58–145)		
Inferonasal					
Mean ± SD	135.96 ± 23.02	130.03 ± 24.12	102.56 ± 21.85	<0.001 *	1–3; 2–3
Median (min–max)	133 (101–197)	127 (84–207)	102(55–165)		
Macular Choroid Thickness					
Average					
Mean ± SD	226.9 ± 21.1	204.1 ± 40.3	186.8 ± 25.7	<0.001 *	1–3
Median (min–max)	225.7 (187–275)	210.9 (123–268)	182.4 (137–227)		
Subfoveal					
Mean ± SD	285.84 ± 17.16	253.13 ± 46.25	223.76 ± 31.30	<0.001 *	1–2; 1–3;2–3
Median (min–max)	283 (248–317)	256.5 (175–324)	217 (172–278)		
Inner Temporal					
Mean ± SD	222.2 ± 26.55	210.27 ± 46.78	206.48 ± 26.58	0.657	
Median (min–max)	224 (180–298)	219 (134–274)	198 (163–254)		
Outer Temporal					
Mean ± SD	210.2 ± 25.2	201.57 ± 49.91	197.16 ± 27.06	0.647	
Median (min–max)	211 (168–263)	211 (114–269)	187 (158–243)		
Inner Nasal					
Mean ± SD	214.44 ± 29.41	185.47 ± 27.22	155.12 ± 26.15	<0.001 *	1–2; 1–3; 2–3
Median (min–max)	221 (116–262)	182.5 (134–248)	158 (108–224)		
Outer Nasal					
Mean ± SD	186.44 ± 25.26	157.83 ± 30.87	127.16 ± 27.34	<0.001 *	1–2; 1–3; 2–3
Median (min–max)	185(143–234)	158.5 (104–240)	124 (80–214)		
Superficial Vessel Density					
Average					
Mean ± SD	43.5 ± 2.9	38 ± 2.8	33.3 ± 1.5	<0.001 *	1–2; 1–3; 2–3
Median (min–max)	43.9 (36–50)	38.2 (31–42)	33.4 (30–36)		
Foveal					
Mean ± SD	23.6 ± 4.8	18.7 ± 2.8	13.7 ± 1.9	<0.001 *	1–2; 1–3; 2–3
Median (min–max)	24.6 (13.5–31.3)	18.4 (13.9–23.8)	14 (9.9–18)		
Inferior					
Mean ± SD	47.5 ± 3.9	42.3 ± 4.8	37.6 ± 2.6	<0.001 *	1–2; 1–3; 2–3
Median (min–max)	48 (38.2–56.3)	42.5 (31.7–49.9)	37.4 (33–44)		
Superior					
Mean ± SD	49.2 ± 3.2	43.1 ± 3.1	38.4 ± 2.1	<0.001 *	1–2; 1–3; 2–3
Median (min–max)	49.6 (42.6–56.4)	44.2 (35.1–48.3)	38.6 (34–42.6)		
Nasal					
Mean ± SD	48.3 ± 2.7	42.6 ± 4.1	38.1 ± 2.3	<0.001 *	1–2; 1–3; 2–3
Median (min–max)	48.7 (42.4–54.1)	43.5 (31.7–48.7)	38.5 (30.5–42.1)		
Temporal				<0.001 *	1–2; 1–3; 2–3
Mean ± SD	48.9 ± 3.1	43.2 ± 3.5	38.5 ± 2.3		
Median (min–max)	49.3 (43–57.6)	43.4 (31.8–48.5)	38.2 (34.7–43.3)		
Deep Vessel Density					
Average					
Mean ± SD	38.3 ± 2.0	37.4 ± 2.6	35.2 ± 1.9	<0.001*	1–3; 2–3
Median (min–max)	37.7 (33–42)	37.6 (30–43)	35.5 (31–39)		
Fovea					
Mean ± SD	18.4 ± 4	17 ± 2.5	12.7 ± 2.6	<0.001 *	1–3; 2–3
Median (min–max)	17.6 (12.5–26.5)	16.5 (12.1–22)	13.3 (6.6–16.8)		
Inferior					
Mean ± SD	43.1 ± 2.3	42.1 ± 4.1	36.7 ± 2.1	<0.001 *	1–3; 2–3
Median (min–max)	43.3 (38.2–46.4)	42.8 (31.6–48.9)	37.5 (31.2–39.6)		
Superior					
Mean ± SD	43.3 ± 2.9	42.6 ± 4.4	42 ± 2.5	0.251	
Median (min–max)	43.2 (36.2–48.2)	42.5 (25.5–48.9)	42.2 (37.7–46)		
Nasal					
Mean ± SD	43.4 ± 2.6	43 ± 3.8	42.9 ± 3.3	0.664	
Median (min–max)	43.2 (38.1–48.2)	43.7 (32.5–48.5)	42.1 (36–48.6)		
Temporal					
Mean ± SD	43.1 ± 2.1	42 ± 3.4	41.8 ± 3	0.339	
Median (min–max)	43.2 (39.6–47)	42.6 (34.6–47.4)	42.1 (35.8–47.5)		

PES: pseudoexfoliation syndrome, PXG: pseudoexfoliative glaucoma, RNFL: retinal nerve fiber layer, SD: standard deviation. * *p* < 0.05; statistically significant.

**Table 3:** Comparison of oxidative stress, inflammatory, and ischemic biomarkers among control, pseudoexfoliation syndrome (PES), and pseudoexfoliation glaucoma (PXG) groups.

	Control (1)	PES (2)	PXG (3)	*p*-Value	
	(*n* = 25)	(*n* = 30)	(*n* = 25)		
Biomarkers					
MDA					
Mean ± SD	12.3 ± 2.4	17.8 ± 5.3	20 ± 3.1	<0.001 *	1–2; 1–3
Median (min–max)	12.8 (7.7–16.4)	18.1 (9.3–34.8)	19.4 (14.7–29.5)		
GSH					
Mean ± SD	0.4 ± 0.1	0.2 ± 0.1	0.2 ± 0.1	<0.001*	1–2; 1–3
Median (min–max)	0.4 (0.2–0.9)	0.2 (0.1–0.4)	0.2 (0.1–0.4)		
IL-6					
Mean ± SD	1.2 ± 1.3	2.9 ± 1.6	1.2 ± 0.7	<0.001 *	1–2; 2–3
Median (min–max)	0.7 (0–4.5)	2.9 (0.6–7.4)	1.1 (0.3–4.1)		
NO					
Mean ± SD	0.1 ± 0	0.1 ± 0	0.1 ± 0	0.821	
Median (min–max)	0.1 (0.1–0.2)	0.1 (0.1–0.2)	0.1 (0.1–0.3)		
iNOS					
Mean ± SD	4 ± 1.5	6 ± 0.8	8 ± 1.1	<0.001 *	1–2; 1–3; 2–3
Median (min–max)	4 (0.6–6.3)	6.2 (3.3–6.8)	8.3 (4.1–8.7)		
Galectin-3					
Mean ± SD	5 ± 2.9	7.3 ± 2	11.7 ± 1.1	<0.001 *	1–3; 2–3
Median (min–max)	4.2 (1–9.8)	7.5 (1.1–11.2)	11.7 (9.4–13.4)		
SCUBE-1					
Mean ± SD	1.4 ± 0.5	1.4 ± 0.7	2.9 ± 0.6	<0.001 *	1–3; 2–3
Median (min–max)	1.5 (0.6–2.2)	1.3 (0.1–3.4)	2.8 (1.4–4.1)		

PES: pseudoexfoliation syndrome, PXG: pseudoexfoliative glaucoma, SD: standard deviation, MDA: malondialdehyde, GSH: glutathione, IL-6: interleukin-6, NO: nitric oxide, i-NOS: inducible nitric oxide synthase, SCUBE-1: signal peptide, CUB domain, and epidermal growth factor-like domain containing protein 1. * *p* < 0.05; statistically significant.

**Table 4:** Correlation of inflammatory, oxidative stress, and ischemic biomarkers with retinal and choroidal parameters in pseudoexfoliation syndrome (PES).

		Age	MDA	GSH	IL-6	NO	iNOS	Galectin-3	SCUBE-1
Retinal Nerve Fiber Layer Thickness									
Average	r	−0.116	0.067	0.267	0.104	−0.060	0.336	0.198	0.140
	*p*	0.542	0.726	0.154	0.585	0.753	0.069	0.295	0.459
Inferior	r	−0.020	−0.111	0.068	−0.210	−0.130	0.144	0.015	0.095
	*p*	0.915	0.561	0.719	0.265	0.494	0.447	0.937	0.619
Superior	r	−0.091	0.179	0.109	0.120	−0.233	0.114	0.076	0.182
	*p*	0.631	0.343	0.565	0.527	0.214	0.549	0.690	0.337
Nasal	r	−0.101	0.145	0.062	0.114	−0.036	−0.453	−0.007	−0.383
	*p*	0.596	0.445	0.745	0.547	0.848	0.012	0.971	0.037
Temporal	r	−0.147	0.020	0.188	−0.001	0.069	0.137	0.048	0.156
	*p*	0.439	0.918	0.319	0.994	0.716	0.470	0.801	0.410
Peripapillary Choroidal Thickness									
Nasal	r	0.157	−0.106	0.065	0.268	0.105	0.072	0.152	0.171
	*p*	0.408	0.577	0.731	0.152	0.582	0.704	0.422	0.365
Superonasal	r	0.151	−0.143	0.089	0.210	0.063	0.124	0.226	−0.040
	*p*	0.425	0.450	0.640	0.264	0.740	0.514	0.231	0.833
Superotemporal	r	0.049	0.168	0.276	0.137	−0.015	0.043	0.344	0.198
	*p*	0.796	0.375	0.140	0.471	0.937	0.821	0.063	0.294
Temporal	r	0.056	0.186	0.182	0.124	0.069	0.087	0.317	0.123
	*p*	0.769	0.325	0.335	0.515	0.718	0.648	0.088	0.518
Inferotemporal	r	0.231	0.085	0.053	0.172	0.050	−0.091	0.189	0.088
	*p*	0.220	0.617	0.783	0.363	0.763	0.633	0.318	0.645
Inferonasal	r	0.016	0.067	0.439	0.109	−0.013	0.094	0.176	0.006
	*p*	0.935	0.725	0.015	0.565	0.956	0.620	0.351	0.976
Macular Choroidal Thickness									
Subfoveal	r	−0.245	0.313	0.441	0.209	−0.183	0.116	0.163	0.044
	*p*	0.192	0.092	0.015	0.268	0.334	0.541	0.389	0.818
Inner Temporal	r	−0.176	0.348	0.415	0.062	−0.040	−0.072	−0.450	0.124
	*p*	0.352	0.060	0.022	0.744	0.816	0.707	0.013	0.513
Outer Temporal	r	−0.196	0.322	0.420	0.026	−0.041	−0.098	−0.457	0.125
	*p*	0.299	0.082	0.021	0.892	0.828	0.606	0.011	0.510
Inner Nasal	r	−0.177	0.236	0.146	−0.268	0.046	0.059	0.102	−0.070
	*p*	0.350	0.209	0.445	0.153	0.809	0.756	0.593	0.711
Outer Nasal	r	−0.102	0.152	0.256	−0.023	0.069	0.002	0.230	0.087
	*p*	0.592	0.423	0.473	0.903	0.717	0.993	0.222	0.648
Superficial Vessel Density									
Foveal	r	0.046	−0.538	−0.063	0.075	−0.080	−0.163	−0.128	0.268
	*p*	0.810	0.002	0.741	0.694	0.674	0.389	0.501	0.153
Inferior	r	0.009	−0.172	0.270	−0.191	−0.172	0.033	0.196	−0.096
	*p*	0.963	0.363	0.150	0.313	0.364	0.863	0.299	0.615
Superior	r	−0.260	−0.008	0.453	−0.057	−0.016	0.110	−0.423	−0.108
	*p*	0.166	0.966	0.012	0.765	0.934	0.563	0.020	0.569
Nasal	r	0.023	−0.350	0.227	−0.113	0.043	0.286	0.297	−0.228
	*p*	0.903	0.058	0.228	0.553	0.823	0.126	0.111	0.226
Temporal	r	0.111	−0.060	0.267	−0.089	−0.065	0.056	0.300	0.043
	*p*	0.560	0.752	0.153	0.642	0.733	0.768	0.107	0.821
Deep Vessel Density									
Foveal	r	0.069	−0.451	0.006	0.045	−0.047	−0.032	−0.181	0.134
	*p*	0.717	0.012	0.973	0.814	0.807	0.866	0.339	0.480
Inferior	r	−0.247	0.304	0.252	−0.068	−0.213	0.061	0.200	−0.056
	*p*	0.188	0.103	0.179	0.721	0.259	0.751	0.288	0.770
Superior	r	−0.295	0.064	0.196	−0.058	0.075	−0.396	−0.398	0.034
	*p*	0.113	0.737	0.298	0.760	0.695	0.030	0.029	0.858
Nasal	r	−0.184	−0.049	0.069	−0.083	−0.099	−0.058	0.133	−0.142
	*p*	0.331	0.796	0.719	0.664	0.602	0.760	0.485	0.455
Temporal	r	−0.130	0.264	0.330	−0.143	−0.182	0.010	0.260	0.097
	*p*	0.495	0.158	0.075	0.451	0.336	0.959	0.166	0.611

**Table 5:** Correlation of inflammatory, oxidative stress, and ischemic biomarkers with retinal and choroidal parameters in pseudoexfoliative glaucoma (PXG).

		Age	MDA	GSH	IL-6	NO	iNOS	Galectin-3	SCUBE-1
Retinal Nerve Fiber Layer Thickness									
Average	r	0.102	0.275	−0.219	−0.145	0.166	−0.358	0.106	0.082
	*p*	0.626	0.183	0.293	0.488	0.429	0.079	0.616	0.697
Inferior	r	−0.151	0.201	−0.096	−0.201	0.082	−0.154	0.035	−0.053
	*p*	0.472	0.335	0.648	0.336	0.696	0.461	0.869	0.803
Superior	r	0.019	0.184	−0.270	0.002	0.240	−0.282	−0.048	−0.120
	*p*	0.930	0.378	0.191	0.992	0.247	0.173	0.821	0.567
Nasal	r	0.001	0.114	−0.311	−0.247	0.260	−0.403	−0.116	0.058
	*p*	0.995	0.587	0.130	0.234	0.209	0.046	0.580	0.784
Temporal	r	0.261	0.285	−0.284	−0.116	0.186	−0.072	0.076	0.263
	*p*	0.207	0.168	0.169	0.580	0.374	0.732	0.717	0.204
Peripapillary Choroidal Thickness									
Nasal	r	−0.311	−0.101	0.411	−0.472	0.264	0.145	0.186	0.015
	*p*	0.131	0.632	0.041	0.017	0.202	0.491	0.372	0.945
Superonasal	r	−0.218	−0.206	0.651	−0.294	0.421	0.110	0.114	−0.001
	*p*	0.296	0.323	0.002	0.154	0.036	0.600	0.587	0.995
Superotemporal	r	−0.088	−0.644	0.419	0.095	−0.088	0.012	0.095	0.080
	*p*	0.674	0.001	0.037	0.651	0.674	0.953	0.652	0.703
Temporal	r	−0.118	−0.318	0.529	0.091	0.342	−0.031	−0.004	−0.040
	*p*	0.575	0.121	0.006	0.674	0.094	0.885	0.986	0.848
Inferotemporal	r	−0.323	−0.128	0.137	0.266	0.445	−0.118	0.012	0.028
	*p*	0.115	0.543	0.514	0.198	0.026	0.573	0.953	0.893
Inferonasal	r	−0.379	−0.153	0.180	0.136	0.624	0.103	−0.089	0.012
	*p*	0.062	0.466	0.390	0.518	0.001	0.623	0.674	0.956
Macular Choroidal Thickness									
Subfoveal	r	−0.287	−0.190	0.200	−0.073	−0.010	−138	0.247	0.032
	*p*	0.165	0.364	0.364	0.727	0.963	0.509	0.234	0.879
Inner Temporal	r	−0.459	−0.311	0.166	−0.089	0.083	−0.243	0.242	0.001
	*p*	0.021	0.130	0.429	0.674	0.694	0.242	0.224	0.996
Outer Temporal	r	−0.448	−0.263	0.268	−0.139	0.184	−0.211	0.258	0.016
	*p*	0.025	0.205	0.195	0.507	0.380	0.311	0.212	0.938
Inner Nasal	r	−0.184	−0.240	0.527	−0.120	0.204	0.078	0.329	−0.014
	*p*	0.379	0.247	0.007	0.568	0.328	0.709	0.108	0.945
Outer Nasal	r	−0.129	−0.327	0.738	−0.152	0.437	−0.179	0.333	−0.021
	*p*	0.538	0.110	0.001	0.468	0.029	0.593	0.104	0.919
Superficial Vessel Density									
Foveal	r	0.043	−0.375	0.188	0.304	−0.479	0.066	0.181	−0.020
	*p*	0.839	0.065	0.368	0.140	0.015	0.753	0.387	0.924
Inferior	r	−0.145	−0.277	−0.020	−0.006	0.091	−0.386	0.100	−0.117
	*p*	0.488	0.180	0.924	0.978	0.665	0.057	0.636	0.578
Superior	r	0.145	−0.571	−0.054	0.137	0.179	−0.298	−0.292	0.189
	*p*	0.488	0.003	0.798	0.514	0.392	0.148	0.157	0.365
Nasal	r	−0.072	−0.372	0.008	−0.024	0.081	−0.624	0.044	0.078
	*p*	0.734	0.067	0.968	0.910	0.702	0.001	0.835	0.711
Temporal	r	0.105	−0.394	0.240	0.163	0.030	−0.305	−0.050	−0.112
	*p*	0.619	0.051	0.248	0.437	0.888	0.139	0.812	0.595
Deep Vessel Density									
Foveal	r	−0.023	−0.481	−0.013	0.421	−0.074	−0.454	0.254	0.055
	*p*	0.912	0.015	0.951	0.063	0.726	0.023	0.221	0.794
Inferior	r	−0.023	−0.434	−0.169	0.250	−0.263	−0.393	−0.129	−0.192
	*p*	0.912	0.030	0.420	0.228	0.203	0.052	0.539	0.357
Superior	r	−0.042	−0.377	−0.122	0.291	−0.044	−0.515	−0.045	−0.168
	*p*	0.843	0.063	0.562	0.158	0.835	0.008	0.829	0.422
Nasal	r	−0.084	−0.076	0.140	−0.212	0.062	−0.077	0.059	0.103
	*p*	0.689	0.718	0.505	0.310	0.769	0.716	0.779	0.623
Temporal	r	−0.086	−0.507	0.073	−0.086	−0.201	0.013	−0.102	−0.108
	*p*	0.683	0.010	0.730	0.682	0.336	0.952	0.629	0.607

**Table 6:** Multivariate regression analysis of inflammatory and oxidative stress markers in relation to retinal and choroidal parameters in pseudoexfoliative glaucoma.

	Coefficients						
Model	Unstandardized Coefficients		Standardized Coefficients			95% Confidence Interval	
Retinal Nerve Fiber Layer Thickness (R^2^: 0.471; *p* = 0.049)							
	β	Standard Error	Β	*t*-Value	*p*-Value	Lower Bound	Upper Bound
Constant	75.949	29.762		2.552	0.02	13.422	138.476
iNOS	−5.816	2.483	−0.561	−2.342	0.031	−11.031	−0.6
SCUBE-1	−0.638	3.286	−0.023	−0.132	0.897	−7.017	8.293
IL-6	−4.877	3.358	−0.319	−1.516	0.146	−12.16	1.951
Galectin-3	−1.201	2.203	−0.119	−0.545	0.592	−5.829	3.426
MDA	1.377	0.715	0.421	1.927	0.07	−0.124	2.878
GSH	4.071	37.232	0.026	0.109	0.914	−74.152	82.293
Peripapillary Choroidal Thickness (R^2^: 0.726; *p* = 0.562)							
Constant	29.153	58.636		0.497	0.625	−129.753	200.075
iNOS	−0.118	4.747	−0.006	−0.025	0.980	−13.390	14.122
SCUBE-1	0.041	7.134	0.001	0.006	0.995	−17.234	23.144
IL-6	−0.053	6.557	−0.002	−0.008	0.994	−14.106	23.111
Galectin-3	0.730	4.300	0.037	0.170	0.867	−10.171	14.238
MDA	3.712	1.575	0.525	2.356	0.060	−1.958	5.960
GSH	−45.056	73.646	−0.150	−0.613	0.547	−299.006	113.616
Macular Choroidal Thickness (R^2^: 0.726; *p* = 0.486)							
Constant	106.463	68.834		1.547	0.139	−33.115	249.174
iNOS	−4.232	5.572	−0.178	−0.759	0.457	−16.717	6.829
SCUBE-1	−0.738	8.374	−0.017	−0.088	0.931	−17.015	17.544
IL-6	−5.765	7.697	−0.157	−0.749	0.464	−23.043	8.810
Galectin-3	7.932	5.048	0.343	1.571	0.134	−2.759	18.132
MDA	2.009	1.849	0.240	1.086	0.292	−1.310	5.467
GSH	−115.145	86.254	−0.324	−1.335	0.199	−288.484	64.665
Superficial Vessel Density (R^2^: 0.662; *p* = 0.002)							
Constant	39.741	2.871		13.844	<0.001	33.710	45.772
iNOS	−0.786	0.232	−0.570	−3.382	0.003	−1.274	−0.298
SCUBE-1	0.016	0.349	0.006	0.045	0.964	−0.718	0.750
IL-6	−0.206	0.321	−0.097	−0.641	0.530	−0.880	0.469
Galectin-3	0.101	0.211	0.075	0.478	0.638	−0.342	0.543
MDA	−0.246	0.077	−0.507	−3.187	0.005	−0.408	−0.084
GSH	3.069	3.597	0.149	0.853	0.405	−4.486	10.626
Deep Vessel Density (R^2^: 0.726; *p* = 0.022)							
Constant	46.218	4.308		10.729	<0.001	37.167	55.269
iNOS	−0.977	0.349	−0.559	−2.802	0.012	−1.710	−0.245
SCUBE-1	0.050	0.524	0.016	0.096	0.925	−1.051	1.151
IL-6	−0.786	0.482	−0.291	−1.633	0.120	−1.798	0.226
Galectin-3	−0.100	0.316	−0.059	−0.316	0.755	−0.764	0.564
MDA	−0.261	0.116	−0.423	−2.251	0.037	−0.504	−0.017
GSH	0.903	5.398	0.034	0.167	0.869	−10.438	12.244

**Table 7:** Diagnostic performance of serum biomarkers in differentiating PXG from controls based on ROC curve analysis.

Biomarker	AUC (95% CI)	*p* Value	Cut-Off	Sensitivity (%)	Specificity (%)
MDA	0.909 (0.846–0.973)	<0.001	>16.36	74.5	100.0
GSH	0.890 (0.822–0.958)	<0.001	≤0.25	70.9	96.0
Galectin-3	0.843 (0.752–0.933)	<0.001	>6.12	92.7	64.0
IL-6	0.766 (0.637–0.895)	<0.001	>0.98	76.4	76.0
iNOS	0.723 (0.609–0.838)	0.001	>5.81	47.3	92.0
SCUBE-1	0.705 (0.594–0.816)	0.003	>2.22	52.7	100.0

AUC: area under curve, CI: confidence interval, MDA: malondialdehyde, GSH: glutathione, IL-6: interleukin-6, iNOS: inducible nitric oxide synthase, SCUBE-1: signal peptide, CUB domain, and epidermal growth factor-like domain containing protein 1.

## Data Availability

The original contributions presented in this study are included in the article; further inquiries can be directed to the corresponding author.
